# Diagnostic performance of T2-weighted CMR for evaluation of acute myocardial injury

**DOI:** 10.1186/1532-429X-14-S1-O62

**Published:** 2012-02-01

**Authors:** Monvadi B Srichai, Ruth P Lim, Narayan Lath, James Babb, Leon Axel, Daniel Kim

**Affiliations:** 1 NYU School of Medicine, New York, NY, USA; 2 Radiology, Singapore General Hospital, Singapore, Singapore; 3 The University of Utah, Salt Lake City, UT, USA

## Summary

We compared the performances of two different fat suppression T2-weighted fast spin echo sequences and demonstrated that T2-weighted CMR has high specificity but overall low sensitivity for detection of myocardial injury. More robust CMR techniques are needed for the detection of acute myocardial injury.

## Background

Edema-sensitive T2-weighted cardiac magnetic resonance (CMR) based on fast spin-echo (FSE) imaging is a useful modality to detect acute myocardial injury. Conventional T2-weighted CMR uses three inversion-recovery (IR) pulses, the first two to null the blood signal, and the last one (STIR) to null fat signal, but suffers from reduced myocardial signal and signal dropout. We compared the performances of two different fat suppression T2-weighted FSE sequences: a) STIR (FSE-STIR) and b) spectral adiabatic inversion recovery fat suppression (FSE-SPAIR) for detection of myocardial injury in patients with cardiomyopathy.

## Methods

65 consecutive patients referred for CMR evaluation of myocardial structure and function underwent FSE-STIR and FSE-SPAIR in addition to cine and late gadolinium enhancement CMR as part of the routine clinical protocol. T2-weighted FSE images were independently evaluated by two readers, blinded to the clinical history and type of sequence, for image quality and artifacts (Likert scale 1-5), and presence of increased myocardial T2 signal suggestive of edema. Disagreements in interpretation of myocardial edema were resolved by consensus. In addition, presence of increased T2 signal suggestive of myocardial edema based on the clinical CMR interpretation, incorporating all CMR sequences available in the study, was also recorded for comparison. Troponin levels acquired during the time of the referral were recorded, and patients with recent (<30 days) troponin elevation greater than 2 times upper limit of normal were diagnosed with acute myocardial injury. The diagnostic performance of each T2-weighted FSE technique for detection of myocardial injury was evaluated.

## Results

There were 22/65 patients (34%) with evidence of acute myocardial injury. Image quality and artifact scores were significantly better with the FSE-SPAIR technique compared to FSE-STIR (2.15 vs 2.68, p<0.01; 2.62 vs 3.05, p<0.01, respectively). The sensitivity, specificity, positive predictive value and negative predictive value are as follows: 27%, 93%, 67%, 71% for FSE-SPAIR, 36%, 91%, 67%, 74% for FSE-STIR and 68%, 98%, 94%, 84% for clinical interpretations. There was a statistically significant difference in sensitivity between the clinical interpretation and each of the T2-weighted techniques, but not between the independent T2-weighted techniques.

## Conclusions

Isolated interpretation of T2-weighted CMR for myocardial edema demonstrates high specificity but overall low sensitivity for detection of myocardial injury. However, real world interpretation in combination with cine and late gadolinium enhancement techniques significantly improves overall sensitivity and diagnostic performance. Newer techniques, such as cardiac T2 mapping, may overcome limitations associated with conventional T2-weighted CMR.

## Funding

1K12HS019473-01 NIH/AHRQ/OEREP.

